# PCB residues in the tissues of sea ducks wintering on the south coast of the Baltic Sea, Poland

**DOI:** 10.1007/s11356-019-04586-4

**Published:** 2019-02-22

**Authors:** Agnieszka Tomza-Marciniak, Bogumiła Pilarczyk, Agata Witczak, Izabella Rząd, Renata Pilarczyk

**Affiliations:** 1 Department of Animal Reproduction Biotechnology and Environmental Hygiene, West Pomeranian University of Technology, Szczecin, Janickiego 29, 71-270 Szczecin, Poland; 2 Department of Toxicology, West Pomeranian University of Technology, Szczecin, Papieża Pawła VI 3, 71-459 Szczecin, Poland; 3 Department of Ecology and Environmental Protection, University of Szczecin, Wąska 13, 71-415 Szczecin, Poland; 4 Department of Ruminant Science, West Pomeranian University of Technology, Szczecin, Janickiego 29, 71-270 Szczecin, Poland

**Keywords:** Biomonitoring, PCBs, TEQ, Sea ducks, Baltic Sea

## Abstract

The Baltic Sea is known to be severely polluted by a range of chemicals, one group of which being PCBs. Although the use and production of PCBs were limited or banned in many countries in the 1970s, their presence is still observed in the environment. The aim of this study was to evaluate PCBs concentration in four species of diving sea ducks, interspecies and tissues differences, and in the case of game species, comparison of the obtained results with maximal residue levels (MRLs) and tolerable weekly intake (TWI). The level of Σi-PCBs was noted in most examined samples (liver, muscle, fat tissue) at levels ranging between < LoD and 2315.45 ng/g lw. The dominant congener was PCB 153, followed by PCB 180 and 138. The mean dl-PCB-TEQ value in the muscles of the tested tufted ducks and common pochards was 0.31 and 0.71 pg-TEQ/g lw, respectively, which is 8–25 and 18–57% of the TEQ maximum limit (ML) value for farm animal muscles. The average decrease in i-ΣPCB concentration in the fat tissue of ducks wintering in the Baltic Sea southern coast was found to be 2.9–3.3%/year. The analysis of PCB residues indicates that the common pochard and tufted duck are not suitable for consumption due to high Σi-PCB concentrations. However, the regular consumption of muscle and liver of game birds does not result in an unacceptable intake of dl-PCBs, i.e., above the TWI value.

## Introduction

The Baltic Sea, being an enclosed inland water area connected to the North Sea by only a few shallow straits, is susceptible to pollution and possesses little self-cleaning ability. The turnover time for the surface water layer varies widely from 1 day to as long as 12 years. However, a complete renewal of the total water mass of the Baltic Sea would take about 25–35 years. This slow renewal results in long-term persistence of hazardous substances in the environment and their accumulation in bottom sediments and hydrobionts (Ducrotoy and Elliott 2008; Helcom 2010). The Helcom (2010) report indicates that polychlorinated biphenyls (PCBs), lead, mercury, cesium-137, DDT/DDE, TBT, benz[a]anthracene, and cadmium are the substances most commonly observed at high contamination ratios (CR, calculated as the ratio of the monitored value of the substance to the threshold value used in the Helcom integrated assessment of hazardous substances), and of these, the highest CRs are demonstrated by PCBs (20%).

The PCBs comprise a group of anthropogenic pollutants which are harmful for human health and the environment. These compounds are resistant to biological and chemical degradation, and being of a lipophilic character, they easily undergo bioaccumulation and biomagnification. PCBs may be transported at long distances through moving air masses, and can potentially pollute regions at great distances from the emission sources (SC 2001). Their harmful activities in relation to humans and animals result from their ability to induce tumors and endocrine disturbances, and weaken reproductive, cognitive, and defensive functions (Carpenter 2006). Therefore, PCBs have been included as one of the 12 contaminants named in the Stockholm Convention in 2001 for persistent organic pollutants (POPs) (SC 2001).

Although the use and production of PCBs were limited or banned in many countries in the 1970s, their presence is still observed in the anthroposphere and the biotic and abiotic parts of the environment (Jepson et al. 2016). Nevertheless, residue levels have begun to decline in both the biotic and abiotic environments since the ban. This decreasing trend has also been reported for the Baltic Sea (Bignert et al. 2008; Nyberg et al. 2015). For instance, Nyberg et al. (2015) report that the concentrations of PCB 153 and PCB 118 in Baltic are now 55–85% lower than in the late 1970s; however, the concentration of PCB 118 in some fish species remains above the target value of 0.024 μg/g lipids (OSPAR 2009). Toxicologically significant levels of PCB have also been found in hydrobionts, mostly within the top trophic levels (Jepson et al. 2016; Law et al. 2012).

The Helcom report (2010) illustrates the regions of Baltic Sea “disturbed by hazardous substances.” Of the areas disturbed by PCBs, the following have been mentioned by name: Bothnian Bay, the Gulf of Riga Western Gotland Basin, the Gulf of Gdansk, the Bornholm and Arkona Basins, Mecklenburg and Kiel Bight, the Belt Sea, and Kattegat. Most areas include wintering locations for many boreal and arctic species of sea duck, which mostly reside in the shallow coastal waters of bays, seas, and estuaries characterized by an abundant food supply: Their diet consists mainly of invertebrates, plants, and small fish (Olney 1963, 1968; Stempniewicz and Meissner 1999). The birds are therefore at risk of exposure to the harmful substances present in the Baltic Sea, including PCBs.

Environmental contamination with PCBs results in them entering the food chain linking animals and people. Their toxic properties, persistence, considerable bioaccumulation potential, and consequent adverse health effects have resulted in the need for the development of food monitoring systems (WHO/FAO 2016). Of particular significance are 12 dioxin-like PCBs (dl-PCBs), which demonstrate similar biological activity to the PCDD/Fs, and 6 common non-dioxin-like PCBs (PCB 28, 52, 101, 138, 153, and 180) which are referred to as indicator PCBs (i-PCBs) by EU Commission Regulation No 1259/2011 (amending Regulation No. 1881/2006).

Food safety assessment is based on maximum residue limits (MRLs) established by EU Commission Regulation No. 1259/2011, while dietary exposure to PCBs is assessed according to tolerable weekly intake (TWI). The TWI for dl-PCBs has been estimated at 14 pg-TEQ/kg bw/week (EFSA 2015). However, the Joint FAO/WHO Expert Committee on Food Additives states that it is not possible to establish TWI values for i-PCBs, particularly due to the limited amount of data and the methodological shortcomings in the performed studies (WHO/FAO 2016).

Estimating the levels of PCBs obtained from consuming game meat is complicated by the fact that the level of consumption of this type of meat and offal is often underestimated, while the families of seabird hunters and fishing enthusiasts can be regarded as a high-consumption subpopulation (Warenik-Bany et al. 2016).

The aim of this study was to evaluate PCBs concentration in four species of diving sea ducks, interspecies and tissues differences, and in the case of game species, comparison of the obtained results with MRLs and TWI.

## Materials and methods

### Material

The study examined four species of duck wintering in the Southern Baltic coast, of which two species were game birds, i.e., *Aythya fuligula* (*n* = 9) and *Aythya ferina* (*n* = 9), and two were protected: *Aythya marila* (*n* = 16) and *Bucephala clangula* (*n* = 8). The chosen species differed with regard to the specific composition of their diets (Table 1).

**Table 1:** Diet characteristics

Species	Protection of species	Diet
Common goldeneye *Bucephala clangula*	Strict legal protection in Poland	Almost entirely animal food, mostly mollusks (*Bivalvia*), crustaceans, water insects (e.g., caddis larvae), small fish and amphibians^a^
Tufted duck *Aythya fuligula*	Hunting season in Poland: 15 August–21 December	Usually animal food (82–97%), mostly mollusks. Small share of plants in the diet (3–18)^b^
Common pochard *Aythya ferina*	Hunting season in Poland: 15 August–21 December	Mostly plant food, especially in spring and summer, and small water animals in autumn and winter—usually insect larvae, and to a smaller extent snails and clams^c^
Greater scaup *Aythya marila*	Strict legal protection in Poland	Mostly mollusks (98%). Small share of plant food in the diet (2%)^a^

^a^Stempniewicz and Meissner (1999)

^b^Olney (1963)

^c^Olney (1968)

**Fig. 1:**
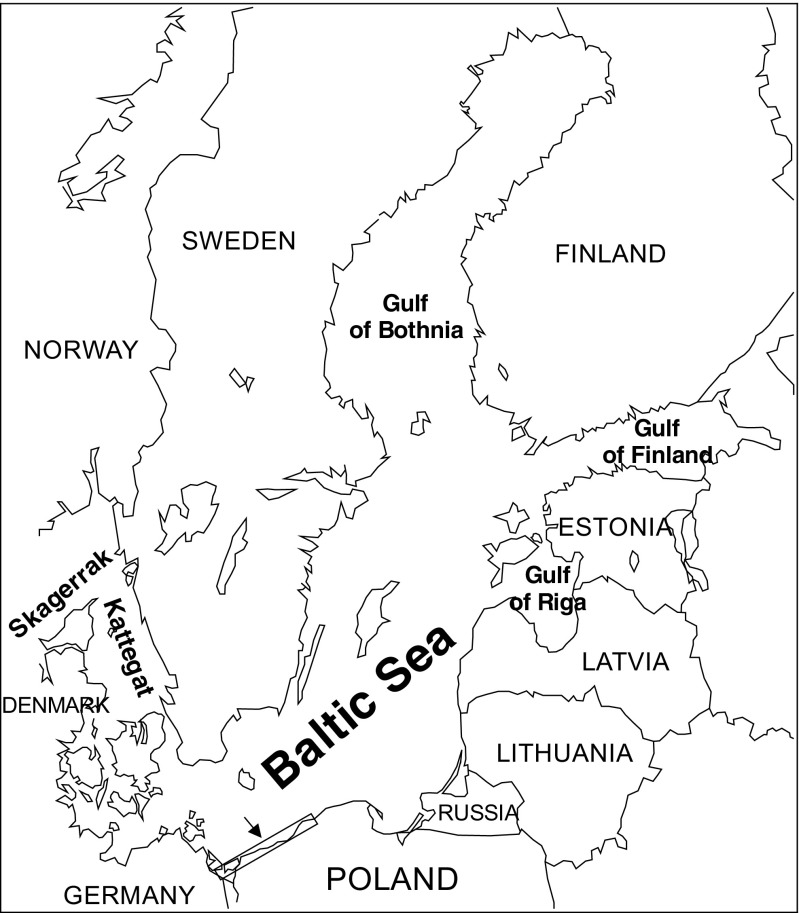
Location of sample collection

As two of the listed species, *A. marila* and *B. clangula*, are legally protected, the examination concerned only dead individuals found in fishing nets, after prior authorization by the Regional Director of Environmental Protection in Szczecin. None of the animals were killed for the purpose of performing the study. All birds were obtained from the same area, i.e., from locations in Poland along the South coast of the Baltic Sea (53°54′37″N, 14°14′49″E to 54°10′32″, 15°35′00″E) during winter 2013 (Fig. 1).

After transport to the laboratory, the physical condition, sex, and age of the birds were evaluated. Due to the fact that within individual species only individual males and juveniles were obtained, the animals were not divided by sex and age in the interpretation of results.

The experimental material included samples of liver, breast muscle, and fat tissue. After section, all tissue samples were homogenized and then stored at − 20 °C until analysis.

### Chemicals

The following reagents were used in the analysis: *n*-hexane, acetone, dichloromethane, and sulfuric acid by Merck (KGaA, Germany), and ethyl ether and anhydrous sodium sulfate by Chempur (Poland). *n*-Hexane and acetone were of HPLC grade. Sulfuric acid (95–97%) and anhydrous sodium sulfate were of *pro analysi* grade. Florisil (60–100 mesh) was obtained from Sigma-Aldrich. The standards for gas chromatography were obtained from AccuStandard, Inc. (New Haven, USA).

### Extraction and clean-up procedure

The samples were analyzed for dioxin-like congeners (dl-PCB number: 77, 114, 118, 126, 156, 157, 169) and non-dioxin-like indicator PCBs (i-PCBs), numbers 28, 52, 101, 138, 153, and 180, according to the EU food legislation (EU Commission Regulation No. 1259/2011). The samples were prepared for analysis according to Tomza-Marciniak et al. (2014). Subsamples of ~ 5–15 g were macerated with anhydrous sodium sulfate in a mortar, until a loose homogenous substance was obtained. The organochlorine compounds were extracted together with lipids using an *n*-hexane/acetone mixture (*v*/*v*, 2.5:1), followed by an *n*-hexane/ethyl ether mixture (*v*/*v*, 9:1). The extracts were then concentrated in a rotary vacuum evaporator at 50 °C to about 2 ml volume and transferred quantitatively with *n*-hexane to 10 ml dried and weighed test tubes.

To determine the percentage of lipids, the dissolvent was evaporated under vacuum in a water bath using a rotary evaporator, and the residue was dried at 80 °C to a solid mass. After determining the lipid mass, the content of the test tubes was dissolved in 2 ml *n*-hexane and purified by adding 8 ml concentrated sulfuric acid. After layer separation, the upper *n*-hexane layer was transferred to an 8-cm^3^ LiChrolut® glass column filled with 1 g activated Florisil. The column was eluted with *n*-hexane and dichloromethane. This eluent was then concentrated to 0.5 ml under vacuum in a water bath.

### Chromatographic analyses

The extract was submitted to gas-liquid chromatographic separation by capillary gas chromatography combined with mass spectrometry (GC/MS) (HP 6890/5973), with an HP-5, 5% phenyl methyl siloxane (30 m × 0.25 mm id × 0.25 μm film thickness) column.

Chromatographic analysis was performed under the following conditions: carrier gas—helium, pressure—12.1 psi; column oven settings—140 °C (0.5 min), increased by 5 °C/min to 200 °C (5 min), increased by 10 °C/min to 280 °C (10 min) and increased by 30 °C/min to 300 °C (1 min). The flow rate (splitless) was 1 cm^3^/min, injection volume 2 μl.

The mass spectrometer transfer line was set to 290 °C and it was operated in the electron ionization (EI) mode at electron energy of 70 eV and a scan range of 50–500 *m*/*z*. The electron multiplier voltage ranged from a 2500 to 3000 V. The electron multiplayer voltage (EMV) was 1624. The full scan modes and selective ion monitoring (SIM) of operation were used in the qualitative analysis and quantification of real samples, respectively.

### Evaluation of the procedure

PCBs were identified and quantified with standard solutions (Custom PCB Standard, AccuStandard, Inc., New Haven, USA).

- Identification of the compounds based on retention time. This process was confirmed by the separation of fortified samples in scan mode, allowing them to be identified based on their individual mass spectra (Table 2). Some of the samples were fortified by adding 200 μl of mixed standard solutions containing 160 ppb (ng/g) of each congener immediately after preparation of the sample.
- The recovery of compounds from internal standards was determined by the addition of a standard solution containing ^13^C (^13^C12-labeled PCB Mixture-A in Nonane, CIL—Cambridge Isotope Laboratorie, Inc. EC-4938) (3,3′,4,4′-TetraCB; 3,4,4′,5-TetraCB; 2′,3,4,4′,5-PentaCB; 3,3′,4,4′,5-PentaCB; 3,3′,4,4′,5,5′-HexaCB; 2,2′,3,4,4′,5,5′-HeptaCB) (50 μl, 120 ppb); Pesticides Surrogate Spike Mix solution (4-8460, SUPELCO, USA), an acetone solution of decachlorobiphenyl (PCB 209) and 2,4,5,6-tetrachloro-*m*-xylene (100 μl, 80 ppb), was used as an internal standard. The internal standard was added to all samples, with one analytical repeat performed in each case with the addition of isotope. The mean non-mono and ortho-PCB congener recoveries were as follows: PCB 77—77.8%, PCB 126—79.6%, PCB 169—87.1%, PCB 114—78.0%, PCB 156—78.1%, PCB 157—82.0%. The efficiency of i-PCB recovery ranged between 77.2 and 88.2%, while that of PCB 209 ranged between 81.1 and 95.3%
- Recovery of compounds from certified reference material: To test the accuracy of the method, a reference material was additionally analyzed with each batch of samples (SRM 1946, National Institute of Standards & Technology, USA). The concentration of PCBs ranged between 80.2 and 90.8% for PCBs in relation to reference values, which indicates that the PCB levels are underestimated (Table 3).
- The quality of the analytical procedure was checked by analyzing a blank sample. A blank sample was included for every ten samples. No cross-contamination was found.

**Table 2:** Parameters of SIM mode for chromatographic analysis of PCBs

PCB	Molecular ion (*m*/*z*)	Confirmation ion (*m*/*z*)
28	256	258; 186
52	256	290; 220
77	292	290; 220
101	326	254; 328
114	326	328; 254
118	326	328; 254
126	326	254; 324
138	360	362; 290
153	360	290; 362
156	360	290; 362
157	360	290; 362
169	360	362; 290
180	394	396; 324

**Table 3:** Validation of the analytical procedure using SRM 1946

PCB congener	Certified/reference concentration (ng/g ww)	Obtained concentration (ng/g ww)	% Recovery
Certified mass fraction			
52	8.1	6.763 ± 1.3	83.2
77	0.327	0.271 ± 0.04	82.9
101	34.6	30.97 ± 0.32	89.5
118	52.1	45.82 ± 1.53	87.9
126	0.38	0.310 ± 0.07	81.6
138	115	101.7 ± 11.6	88.4
153	170	154.4 ± 8.71	90.8
156	9.52	7.635 ± 0.54	80.2
169	0.106	0.088 ± 0.04	83.2
180	74.4	61.75 ± 3.27	83.0
Reference mass fraction			
28	2	1.69 ± 0.15	84.7

The limit of quantification (LOQ) was determined as the concentration of an extract that produced an instrumental response for two different tested ions with a signal-to-noise ratio of 3:1 for the less-sensitive signal (Commission Regulations (EU) No. 589/2014). The LOQ for the analyzed compounds was 0.02 ng/g ww. The toxic equivalents (TEQs) were calculated for dioxin-like PCBs (WHO-dlPCB-TEQ) using the toxic equivalency factor (TEF) values described by Van den Berg et al. (2006).

### Dietary intake assumption

Little data on the consumption of wild duck liver and muscle is available in Europe; therefore, the weekly intake was estimated based on the assumption of long-term consumption by adults and children, with respective body masses of 70 and 23 kg (Warenik-Bany 2016). The consumers were separated into three groups based on different proposed consumption scenarios: (a) those often consuming meat and offal from game birds (24 times a year), (b) those consuming meat and offal from game birds from time to time (six times a year), (c) those rarely consuming meat and offal from game birds (twice a year). The portion size of meat was assumed as 200 g for an adult and 100 g for a child, and the portion size of liver as 120 g for adults and 60 g for children.

### Statistical analysis of the data

Statistical analysis of the data was performed using Statistica software (StatSoft Inc., ver. 12 StatSoft, Tulsa, OK, USA). Prior to the analyses, the distribution of the data was determined using the Shapiro-Wilk *W* test. As the data did not follow a normal distribution, non-parametric analytical procedures were used. The significance of differences was analyzed using the Kruskal-Wallis test and the Mann-Whitney *U* test. Differences were considered as significant at *p* < 0.050. The correlations between PCB concentration and lipid content of liver and muscle tissue were determined by Spearman’s rank correlations (rs). All results were given as arithmetic means and standard deviations (Sd).

## Results and discussion

### Status of contamination

The i-PCBs concentrations in the tissues of sea ducks wintering in the Baltic Sea are given in Table 4. Their presence was noted in most of the examined samples, at levels ranging from < LoD (e.g., tufted duck fat tissue) to 2315.45 ng/g lipid weight (lw) (Σi-PCB concentration in tufted duck liver). In muscle, liver, and fat tissue, PCB 153 was the dominant congener, reaching 36–49% Σi-PCBs, followed by PCB 180 and 138. The large share of PCB 153 with regard to the total amount of tested indicator PCBs (Σi-PCBs) can be attributed to the fact that it is uniquely resistant to the metabolic changes taking place in animals (Grimm et al. 2015; Pikkarainen 2007), hence favoring its accumulation in tissues, and that it is widely prevalent in the Baltic Sea (Helcom 2010; OSPAR 2005). Pikkarainen (2007) found the concentration of PCB 153 in bivalves to range from 29 to 40 ng/g lw, which is about 10% of the concentration found in the muscle tissue of mollusk-feeding birds apart from the common pochard. Additionally, the PCB 153 concentrations observed in fish and mollusks of the Baltic Sea were found to be a few times higher than the threshold level (2.5 ng/g lw), and higher values have been noted in Little Belt, southern parts of the Kattegat (Denmark), Szczecin Lagoon (Poland), southern parts of the Bothnian Sea, and in Bothnian Bay (Sweden/Finland) (Helcom 2010; OSPAR 2005).

**Table 4:** Concentration of indicator PCBs, PCB 118, and Σi-PCBs in seabirds

Species	Concentration, ng/g lw							
	PCB 28	PCB 52	PCB 101	PCB 153	PCB 138	PCB 180	Σi-PCBs	PCB 118
Common goldeneye—*Bucephala clangula*								
Muscle								
Mean	9.11	2.09	3.18	398.04	245.01	296.44	953.87 ^A^	119.73
Sd	2.53	2.48	3.23	153.03	195.13	265.38	586.53	99.07
Min	6.73	< LOQ	< LOQ	173.49	68.49	88.63	339.34	32.42
Max	12.68	4.89	7.56	517.30	523.86	685.77	1752.06	262.21
Liver								
Mean	2.65	5.46	2.17	440.80	243.37	212.34	906.78	108.25
Sd	3.94	3.84	1.52	413.71	212.82	163.98	791.64	69.98
Min	0.00	0.66	0.00	16.62	13.51	12.99	43.78	17.84
Max	8.35	10.05	3.22	987.18	512.36	413.38	1923.98	188.59
Fat tissue								
Mean	5.68	4.18	3.42	265.36	176.91	190.63	646.18	42.60
Sd	1.47	5.06	1.24	121.08	60.95	153.15	288.40	7.49
Min	4.02	0.98	2.01	131.21	119.02	88.01	347.18	37.93
Max	6.81	10.01	4.32	366.54	240.51	366.66	922.65	51.24
Tufted duck—*Aythya fuligula*								
Muscle								
Mean	5.59	6.30	1.86	231.14	192.41	148.96	586.27	65.25
Sd	3.30	3.09	2.04	189.59	113.62	82.57	358.41	21.55
Min	0.00	1.18	0.00	24.98	24.33	23.39	78.82	32.13
Max	7.68	8.99	4.93	420.29	317.68	234.36	956.81	88.09
Liver								
Mean	7.14 ^a^	4.85	6.28	658.95	269.45	435.87 ^A,B^	1382.54	124.74
Sd	8.26	4.58	6.11	391.59	172.64	295.38	854.06	84.88
Min	0.00	0.00	1.02	157.66	53.03	69.91	281.63	28.99
Max	14.90	11.05	14.97	1114.07	436.18	733.48	2315.45	235.35
Fat tissue								
Mean	2.2 ^a^	0.6	1.4	72.3	52.1	63.2	191.80	33.20
Sd	2.1	0.7	1.7	57.5	43.3	49.3	130.80	31.98
Min	< LOQ	< LOQ	< LOQ	< LOQ	< LOQ	< LOQ	< LOQ	< LOQ
Max	4.7	1.5	3.4	138.2	105.5	119.0	293.96	76.92
Common pochard*—Aythya ferina*								
Muscle								
Mean	6.52	3.46	4.37	243.01	141.61	164.42	563.39 ^A^	13.01 ^a^
Sd	4.59	3.20	3.64	66.22	45.75	47.29	86.06	15.00
Min	1.31	0.00	1.37	108.31	88.94	116.71	378.84	0.86
Max	13.94	8.81	10.52	324.61	222.88	231.95	641.02	42.21
Liver								
Mean	10.59 ^a^	5.90 ^a^	7.25 ^a^	348.82 ^a^	174.55 ^a^	220.84 ^a,B^	767.93	12.45 ^b^
Sd	5.78	7.46	7.13	208.04	65.63	68.32	295.12	12.03
Min	1.55	< LOQ	< LOQ	77.88	52.28	76.34	223.56	1.02
Max	17.06	22.68	21.82	739.73	264.56	284.49	1278.31	33.25
Fat tissue								
Mean	2.01 ^a^	0.47 ^a^	1.58 ^a^	124.10 ^a^	66.96 ^a^	70.93 ^a^	266.05	26.41 ^a,b^
Sd	0.85	0.34	0.42	41.51	21.21	45.68	39.05	3.48
Min	1.31	0.00	1.17	85.55	46.54	35.03	207.89	22.22
Max	3.25	0.79	2.13	182.07	96.31	135.30	290.77	30.69
Greater scaup—*Aythya marila*								
Muscle								
Mean	2.34	1.94	2.44	175.48	97.52	92.82	372.53	35.60
Sd	2.76	2.38	2.78	153.87	79.11	81.21	314.28	28.70
Min	< LOQ	< LOQ	< LOQ	6.47	3.14	5.15	14.77	1.63
Max	6.61	6.89	9.23	506.99	237.48	265.52	1024.23	99.76
Liver								
Mean	3.72	3.25	3.27	171.88	110.60	91.86 ^A^	384.57	41.96
Sd	3.37	2.70	3.34	178.08	91.74	114.57	368.38	47.84
Min	< LOQ	< LOQ	< LOQ	9.64	4.64	3.37	17.65	6.55
Max	9.18	8.70	10.07	503.49	271.30	407.54	1186.19	158.30
Fat tissue								
Mean	5.05	5.43	2.78	213.39	194.89	174.29	595.82	61.18
Sd	3.26	6.49	1.72	124.901	148.42	142.20	387.78	40.23
Min	0.31	< LOQ	0.17	16.23	8.80	8.48	34.19	2.80
Max	9.86	16.50	5.36	335.79	490.95	371.00	1222.96	135.88

(a,…) The same lowercase letters denote significant (*p* < 0.05) intraspecies differences between the tissues. (A,…) The same capital letters denote significant (*p* < 0.05) interspecies differences

A similar profile of the analyzed compounds, i.e., the share of individual congeners in Σi-PCBs, was observed by Guruge et al. (2001) in oceanic birds from the North Pacific and Southern Ocean: Regardless of species and study area, the highest peak was observed for PCB 153, followed by PCB 180, 138, and 118. Similar results were also obtained by Naso et al. (2003), Klasson-Wehler et al. (1998), and Mora (1996).

The concentrations of low-chlorinated congeners (up to five chlorine atoms per molecule) ranged from undetectable, e.g., PCB 52 and 101 in the muscle of the common goldeneye (Table 4), to 22.68 ng/g lw, observed for PCB 52 in common pochard liver. The concentration of high-chlorinated congeners (more than five chlorine atoms in the molecule) was much higher, ranging from tens of nanograms per gram lw to more than 1000 ng/g lw (observed for PCB 153 in tufted duck liver). This congeneric profile is typical of this sample type, i.e., animal tissues, and results mostly from the susceptibility of the examined compounds to processes related to their metabolism. For example, PCB 180, 153, 138, and 118 are more resistant to metabolic cytochrome P450-mediated attacks due to a lack of unsubstituted adjacent meta- and para-positions on the biphenyl rings. In contrast, PCB 28, 52, and 101 are more rapidly metabolized and excreted by animals due to their free adjacent ortho-meta or meta-para positions on the rings (Oliver and Niimi 1988; Oberg et al. 2002).

**Fig. 2:**
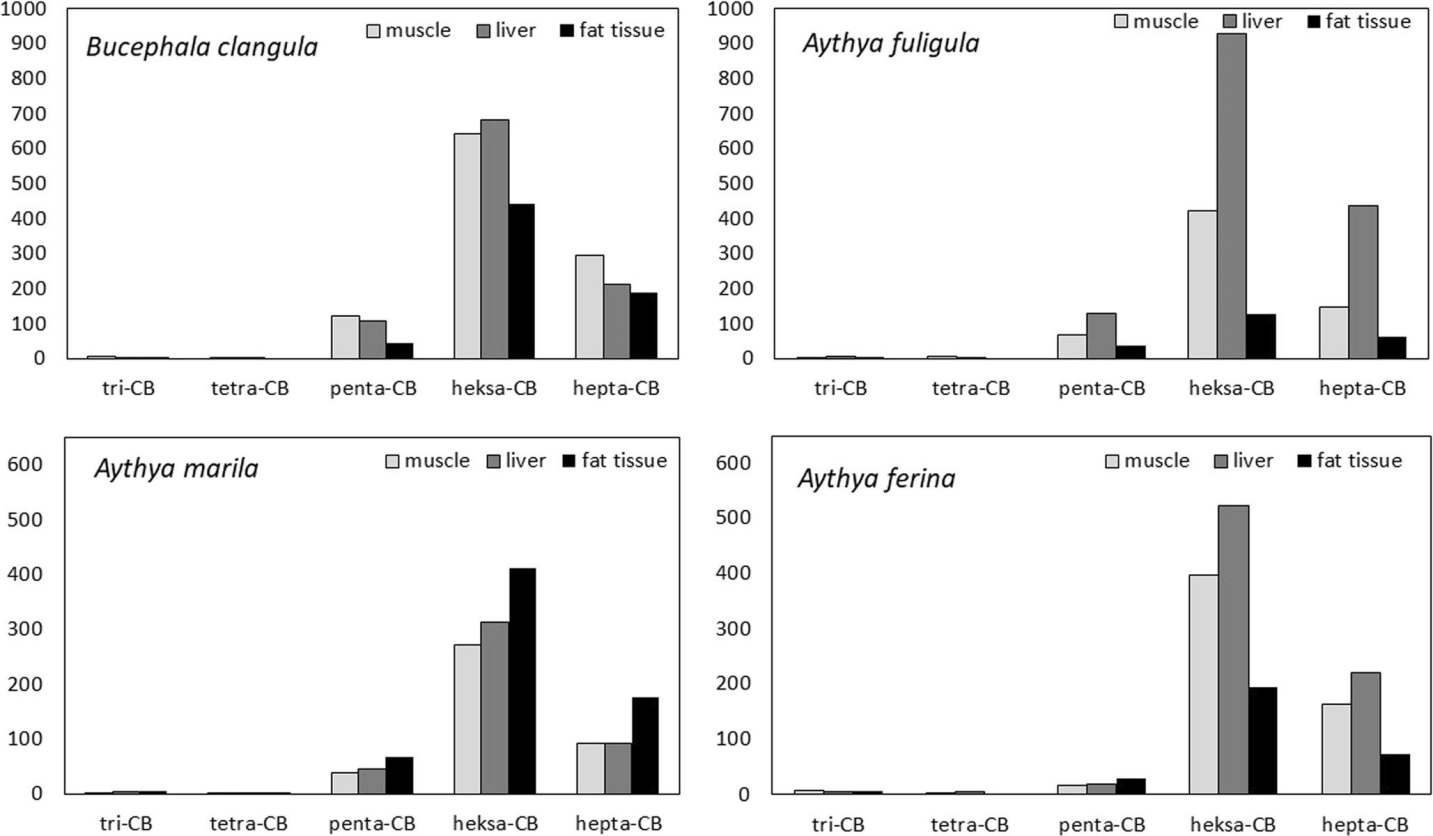
The concentrations (mg/kg lw) of homologs in the examined seabird tissues

The concentrations of particular homologs in the examined seabird tissues are shown in Fig. 2. The homolog classes in the muscle, liver, and fat tissue were found to be dominated by hexa- and heptabiphenyls in all tested species, with these present at concentrations many times higher than those of the tri-, tetra, and pentachlorobiphenyls. However, it has been reported that penta-, hexa-, and heptachlorobiphenyls predominate in birds from the Southern Ocean, while those from the North Pacific display a greater share of pentachlorobiphenyls than heptachlorobiphenyls (Guruge et al. 2001). This situation may be attributed to the fact that the Baltic Sea and the Southern Ocean are much both more exposed to PCB cocktails containing highly chlorinated congeners than the North Pacific. The differences observed between basins result mainly from the global distillation of PCBs: low-chlorinated congeners volatilize in warm regions and are transported to colder ones, while highly chlorinated compounds remain closer to the sources of emission (Wania and Mackay 1996). This has been confirmed by Mai et al. (2016) which reported that while higher-chlorinated PCBs have been found to be present in the air near the shoreline, more volatile congeners tend to be observed in the open waters far from shore. Significant roles are also played by the concentration of point sources of PCB emission along the shoreline of the water body. Wania et al. (2001) found the main source of PCBs for the Baltic to be atmospheric deposition, which predominates over the process of volatilization.

The mean ∑i-PCB concentration observed in the fatty tissue and liver of common goldeneye, tufted duck, common pochard, and greater scaup from the Baltic region was found to be lower than in analogous organs obtained previously from albatrosses living in the North Pacific or from glaucous gulls in Svalbard (Table 5). These differences seem to be high enough that the underestimation of PCBs (Table 3) in these studies and another set of analyzed congeners by the cited authors (Guruge et al. 2001; Skaare et al. 2000) should not be very important.

**Table 5:** PCBs concentration detected in water birds from different regions

Species	Location	Years	Organ/ tissue	PCDD/F-dlPCB pg-TEQs/g	PCBs ng/g	Reference
Surf scoters *Melanitta perspicillata*	South coast of British Columbia, Canada	1998–2001	Liver	7.1	13.3 (ww) ∑52 congeners	Wilson et al. (2010)
Northern pintail *Anas acuta*	Caspian Sea	2008	Liver	–	29.5 (ww) ∑7 congeners	Rajaei et al. (2010)
Eurasian teal *Anas creaca*					11.5 (ww) ∑7 congeners	
Mallard *duck* *Anas platyrhynchos*					29.5 (ww) ∑7 congeners	
European herring gull *Larus argentatus*	Southern Baltic	2010–2012	Muscle	206.5	–	Falkowska et al. (2016)
			Liver	711.9		
Black-footed albatross *Phoebastria nigripes*	North Pacific	1993–1998	Fat	–	72,600 (ww) ∑14 congeners	Guruge et al. (2001)
Laysan albatross *Phoebastria immutabilis*				–	18,900 (ww) ∑14 congeners	
Royal albatross *Diomedea epomophora*	Southern Ocean			–	2300 (ww) ∑14 congeners	
Black-browed albatross *Thalassarche melanophris*				–	910 (ww) ∑14 congeners	
Giant petrel *Macronectes* spp.	Southern Ocean	2011	Blood		6.10–72.2 (ww) ∑21 congeners	Roscales et al. (2016)
American white pelican *Pelecanus erythrorhynchos*	Canada	1989	Liver	–	A: 493 (ww) B: 3195 (ww) ∑42 congeners	Donaldson and Braune (1999)
Glaucous gull *Larus hyperboreus*	Norway Svalbard	1991–1998	Liver	–	16,000 (lw) n/d	Skaare et al. (2000)
Common eider *Somateria mollisima*	Norway Sklinna Røst	2012	Eggs			Huber et al. (2015)
					10.9 (ww) 76.9 (ww) ∑32 congeners	
Black cormorants *Phalacrocorax carbo sinensis*	Poland		Liver Muscle		13,000–69,000 (lw) 19,000–110,000 (lw) n/d	Falandysz et al. (2003)
Least tern *Sternula antillarum*	Coastal Georgia, USA	2011–2012	Whole bodies of chicks Eggs		418–3299 (dw) 544–4758 (dw) ∑12 congeners	Robinson et al. (2015)
Skua *Stercorarius antarcticus*	Antarctic King George Island	2010–2011	Muscle		54.100* (dw) ∑14 congeners	Wolschke et al. (2015)
Kentish Plovers *Charadrius alexandrines*	Bohai Bay China	2015–2016	Liver Muscle Eggs		101 (lw) 113 (lw) 56.3 (lw) ∑19 congeners	Zheng et al. (2018)

*A* female, *B* male, *ww* wet weight, *lw* lipid weight, * Σdl-PCB, *n/d* no data

Comparing the results presented by different authors is difficult due to the different ways of expressing results, i.e., the wet weight (ww) or the weight of lipids (lw). Assuming the typical lipid content in the muscle, liver, and adipose tissue of wild birds to be 1.5, 5, and 30%, respectively, the results can be converted from ww to lw by using respective conversion factors of 67, 20, and 3 for each tissue type.

Guruge et al. (2001) reported that PCBs were found to be present at mean concentrations of 72,600 and 18,900 ng/g ww in the fatty tissue of black-footed albatross and laysan albatross taken from the North Pacific, and 16,000 ng/g lw in the liver tissue of a glaucous gull from Norway (Skaare et al. 2000). In the present studies, the maximal observed concentrations of ∑i-PCBs in bird liver and fat tissue were 2315.45 and 1222.96 ng/g lw, respectively.

Research conducted in various regions of the world indicates that PCBs are still present in the marine environment (Falkowska et al. 2016; Huber et al. 2015; Robinson et al. 2015; Roscales et al. 2016; Wolschke et al. 2015; Zheng et al. 2018) (Table 5). Wilson et al. (2010) and Rajaei et al. (2010) reported that the liver of birds from South coast of British Columbia, Canada and Caspian Sea contained 10–30 ng PCBs/g ww. For comparison, the concentration of PCBs in eggs of Common eider from Røst, Norway was 2-fold higher: 76.9 ng/g ww (Huber et al. 2015). Similar concentration of PCBs was observed in whole blood of Giant petrel from the Southern Ocean Roscales et al. (2016) (Table 5). Brown et al. (2018) investigated 62 PCB congeners in Common loons from Alberta, Canada, and the level of these compounds in whole blood ranged from 2.48 to 167 ng/g ww. Higher concentration of PCBs was found in the liver of American white pelican from Canada, > 490 ng/g ww (Donaldson and Braune 1999). PCBs were quantitated also in Black harriers from South Africa, and the concentration in blood plasma ranged from 0 to 6.93 ng/ml and 0–13.74 ng/ml in adults and nestlings, respectively (Garcia-Heras et al. 2018).

The calculated ∑PCB values were compared with thresholds established by previous toxicity studies in all four species. The harmful effects of PCBs of birds include immune suppression, reduced hatchability, decreased fertility, embryonic deformities, disruption of growth, and in extreme cases, mortality (Barron et al. 1995; Eisler 1986). Custer and Heinz (1980) report a no observed effect concentration (NOEC) of PCBs for mallards of 29.5 μg/g ww of carcass, while Koeman et al. (1973) report NOEC values of 319 μg/g ww of liver and 269 μg/g ww of carcass in great cormorants, with adult mortality as the chosen parameter. Regarding the effect of PCBs on the immune system, it was found that in the glaucous gull, a concentration higher than 15–29 μg/g ww causes immunosuppression and increases susceptibility to parasite infections (Naso et al. 2003). In turn, a study on American kestrels found the LOEC value for a decrease in male fertility to be 91.6 μg/g lw (≈ 4.5 μg/g ww) of liver tissue (Bird et al. 1983).

The PCB concentrations observed in birds from the southern Baltic Sea are much lower than those given above.

### Differences between organs

Tables 4 and 6 present the concentrations of ∑i-PCB in the organs of the tested birds according to lipid and wet weights, respectively, including statistically significant differences between organs. Significant (*p* < 0.05) differences in levels of PCB residues expressed in lipid weight between muscles, livers, and fat tissue were observed in only a few cases (Table 4). No significant differences between organs were observed in the common goldeneye and greater scaup, while the concentrations of some indicator congeners were higher in the liver tissue than fat tissue in the tufted duck and common pochard. Also, Sakellarides et al. (2006) observed that organochlorine concentrations were greater in the liver than the fat tissues of waterbirds. Such a situation may be caused by a redistribution of PCBs from fat tissue to the muscles and liver, which has been observed in conditions such as starvation (de Freitas and Norstrom 1974).

**Table 6:** Concentration of Σi-PCBs in wet weight of birds’ tissues

Species	Concentration of Σi-PCBs (ng/g ww)		
	Muscle	Liver	Fat tissue
Common goldeneye	8.166 ± 6.401^a^	14.53 ± 9.324	195.6 ± 57.32^a^
Tufted duck	7.424 ± 5.162^a^	18.51 ± 12.57^b^	181.8 ± 123.6^ab^
Common pochard	8.926 ± 6.037^A^	11.54 ± 6.497	101.7 ± 24.71^A^
Greater scaup	7.019 ± 5.162^a^	7.501 ± 6.055^A^	168.0 ± 116.7^aA^

Small letters indicate statistically significant differences at *p* ≤ 0.05. The capital letters indicate statistically significant differences at *p* ≤ 0.001

The ∑i-PCB concentration was found to be significantly lower in muscle tissue than fat tissue in all tested bird species with regard to wet weight (*p* < 0.05). However, no significant difference was found between liver and muscle tissue (*p* < 0.05).

### Interspecies differences

Variations in PCB concentrations, as well as in the age, sex, and species of the bird, may influence the specific toxicokinetics and intake of congeners, differential feeding behavior, or PCB elimination associated with maternal transfer. Dietary PCBs are rapidly and extensively absorbed by birds, and are internally distributed according to the lipid content of the tissue (de Freitas and Norstrom 1974).

The examined species of sea ducks demonstrated comparable concentrations of i-PCBs (Table 4). Although the ∑i-PCB level in the fat tissue was two to three times lower in the tufted duck and common pochard than in the common goldeneye, these differences were not statistically significant (*p* < 0.05). In the case of muscle tissue, the ∑i-PCB level was highest in common goldeneye (953.87 ng/g lw). However, although this concentration was much higher than observed in the common pochard (563.39 ng/g lw), this difference was again not statistically significant (*p* < 0.05). The most likely cause was the large standard deviation of the obtained results, which in turn may be due to the relatively small numbers of species members included in the study. However, it was not possible to obtain a larger number of animals in the time available.

The common pochard favors a plant-based diet. It is possible that birds whose food base consists mostly of plants will be characterized by lower residues of PCBs in comparison to birds which feed on other animals, especially mollusks. However, no significant differences were observed between PCB residues in the common pochard and other seabird species in the present study. An exception is the hepatic PCB 180 concentration, which was significantly (*p* < 0.05) lower in the common pochard than the tufted duck.

Our findings indicate that younger and weaker ducks, possessing smaller fat deposits, display higher PCB residues in the lipid fraction than ducks characterized by higher amounts of fat tissue. Lincer and Peakall (1973) found that an increase in PCB concentration in tissues is associated with insufficient food supply, and attribute this to the mobilization and consumption of fat for metabolic purposes.

### Assessment of consumer exposure to PCBs

Of the four examined species, the tufted duck and common pochard are species of game bird. Our study has shown that their tissues are characterized by large amounts of i-PCBs, especially the muscle and liver tissue. The observed concentrations and Σi-PCB levels exceeded the MRLs established for the tissues of farm animals by many times (Table 7). In fact, in no case was the concentration of Σi-PCBs lower than the MRL value, indicating that these organs are not suitable for consumption. A complete assessment of the possible risk to consumers associated with the consumption of i-PCBs could be made by comparing estimated weekly intake (EWI) with the TWI. The EWI of Σi-PCBs and dioxin-like PCBs from muscle and liver of two game ducks based on the three consumption scenarios described earlier is given in Table 8. The i-PCB data indicates that twice the amount of Σi-PCBs is obtained from the muscle and liver of tufted duck than common pochard, irrespective of consumption scenario. For adults, the median intake of Σi-PCBs varied from 0.396 (muscle of common pochard) to 13.51 ng/kg bw/week (liver of tufted duck). For children, the median intake of i-PCBs was greater, and varied from 0.312 to 20.27 ng/kg bw/week. Unfortunately, a further analysis is not possible, as the TWI has not been established for this group of compounds (WHO/FAO 2016).

**Table 7:** Comparison of study results to normative values

	Concentration					
	TEQ (pg/g lw)			i-ΣPCBs (ng/g lw)		
	Liver^a^	Muscle^a^	Fat^a^	Liver	Muscle	Fat
This study						
Common goldeneye	0.23 ± 0.06 2% of tTEQ	0.18 ± 0.19 5–14% of tTEQ	0.07 ± 0.06 2–6% of tTEQ	906.78 < MRL = 0%	953.87 < MRL = 0%	646.18 < MRL = 0%
Common pochard	0.29 ± 0.26 3% of tTEQ	0.31 ± 0.15 8–25% of tTEQ	0.12 ± 0.10 3–10% of tTEQ	767.93 < MRL = 0%	563.39 < MRL = 0%	266.05 < MRL = 0%
Tufted duck	0.83 ± 1.27 8% of tTEQ	0.71 ± 0.72 18–57% of tTEQ	0.11 ± 0.14 3–9% of tTEQ	1382.54 < MRL = 0%	586.27 < MRL = 0%	191.80 < MRL = 0%
Greater scaup	0.19 ± 0.15 2% of tTEQ	0.13 ± 0.17 3–10% of tTEQ	0.10 ± 0.08 2.5–80% of tTEQ	384.57 < MRL = 6%	372.53 < MRL = 12%	595.82 < MRL = 6%
Commission Regulation (EU) No. 1259/2011						
Farm animal liver	10^b^	–	–	40	–	–
Farm animal meat	–	1.25–4^b^	–	–	40	–
Farm animal fat			1.25–4^b^	–	–	40

^a^dl-PCB-TEQ

^b^tTEQ = PCDD/F-dlPCB-TEQ

**Table 8:** Estimated weekly intake of non-dioxin-like PCBs (Σi-PCBs) and dioxin-like PCBs from muscle and liver of two hunting ducks based on consumption scenario

Species		Meal percentile	PCBs level	Weekly intake of PCBs per kg bw					
				Frequent consumers (24/year)		Periodic consumers (6/year)		Occasional consumers (2/year)	
				Adult	Children	Adult	Children	Adult	Children
Σi-PCBs (ng/g ww)									
Tufted duck									
Muscle									
		P50 P97.5	7.746 13.66	10.07 17.76	15.49 27.32	2.556 4.508	3.887 6.830	0.852 1.503	1.317 2.322
Liver									
		P50 P97.5	16.89 40.65	13.51 32.52	20.27 48.78	3.378 8.130	5.067 12.20	1.182 2.846	1.689 4.065
Common pochard									
Muscle									
		P50 P97.5	8.441 19.87	10.97 25.83	16.88 39.74	2.785 6.557	4.220 9.935	0.928 2.186	1.435 3.378
Liver									
		P50 P97.5	11.50 22.87	9.200 18.30	13.800 27.44	2.300 4.574	3.450 6.861	0.805 1.601	1.150 2.287
dl-PCB-TEQ (pg/g ww)									
Tufted duck									
Muscle									
		P50	0.004	0.005 (0.04% of TWI)	0.008 (0.06% of TWI)	0.001 (0.007% of TWI)	0.002 (0.01% of TWI)	0.0004 (0.003% of TWI)	0.0007 (0.005% of TWI)
		P97.5	0.011	0.014 (0.1% of TWI)	0.022 (0.16% of TWI)	0.004 (0.03% of TWI)	0.006 (0.04% of TWI)	0.001 (0.007% of TWI)	0.002 (0.01% of TWI)
Liver									
		P50	0.010	0.008 (0.06% of TWI)	0.012 (0.09% of TWI)	0.002 (0.01% of TWI)	0.003 (0.02% of TWI)	0.0001 (0.001% of TWI)	0.001 (0.007% of TWI)
		P97.5	0.016	0.013 (0.09% of TWI)	0.020 (0.14% of TWI)	0.003 (0.02% of TWI)	0.004 (0.03% of TWI)	0.001 (0.007% of TWI)	0.002 (0.01% of TWI)
Common pochard									
Muscle									
		P50	0.005	0.007 (0.005% of TWI)	0.010 (0.07% of TWI)	0.002 (0.01% of TWI)	0.003 (0.02% of TWI)	0.0006 (0.004% of TWI)	0.001 (0.007% of TWI)
		P97.5	0.011	0.014 (0.01% of TWI)	0.022 (0.16% of TWI)	0.004 (0.03% of TWI)	0.006 (0.04% of TWI)	0.001 (0.007% of TWI)	0.002 (0.01% of TWI)
Liver									
		P50	0.009	0.007 (0.005% of TWI)	0.011 (0.08% of TWI)	0.002 (0.01% of TWI)	0.003 (0.02% of TWI)	0.0006 (0.004% of TWI)	0.0009 (0.006% of TWI)
		P97.5	0.013	0.011 (0.08% of TWI)	0.016 (0.11% of TWI)	0.003 (0.02% of TWI)	0.004 (0.03% of TWI)	0.0009 (0.006% of TWI)	0.001 (0.007% of TWI)

In this study, total TEQ values were calculated based on seven dioxin-like PCBs. Assuming that dl-PCBs account for 60 to 85% of the total TEQ concentrations reported in birds (Kannan et al. 2002), the obtained results are alarming. The mean dl-PCB-TEQ value in muscles of tufted duck and common pochard were 0.31 and 0.71 pg-TEQ/g lw, respectively, which correspond to 8–25 and 18–57% of the TEQ set for farm animal muscles.

The main factor affecting the relatively high TEQ value was the fact that of all dl-PCBs, the dominant congener was PCB 126, which had the highest TEF value. Similarly, PCB 126 was found to be the dominant dl-PCB in the livers in a study of surf scoters from the southern coast of British Columbia (Wilson et al. 2010). However, the EWI value calculated for the three proposed consumption scenarios was low, and in no case did it exceed the TWI value, set at 14 pg-TEQ/kg/bw. The highest EWI value only reached 0.16% of TWI, observed for tufted duck meat (97.5 percentile of dl-PCBs level) for children in the frequent consumption scenario (24 times per year).

Different results were obtained in a study in Poland by Warenik-Bany et al. (2016), who examined the uptake of dioxins from the liver and muscle of Cervidae. They found that in the case of liver, the intake values for frequent adult and children consumers may reach almost 300 and 700% of TWI, respectively. Even though only dl-PCBs were examined in the present study, excluding PCDD/Fs, the differences in intake values nevertheless appear to be rather large.

### Temporal differences in PCB residues

In the case of birds, PCB bioaccumulation is affected by sex, age, and diet, but also by the time of residence in PCB-contaminated areas. Studies by Falandysz and Szefer (1988) showed that the content of PCBs increased in ducks during the wintering period in the southern Baltic Sea. However, for many years, the content of permanent organic pollutants, including PCBs, has been decreasing in the Baltic Sea, which is related to limitations in the use and production of these substances. PCB concentration has also been decreasing over time in bottom sediments, mollusks, and various fish species (Bignert et al. 2008; Nyberg et al. 2015; Pikkarainen 2007). Bignert et al. (2008) report the annual decrease in concentration of these compounds to be about 5–10%, beginning in the late 1970s. In turn, Nyberg et al. (2015) estimate a decrease in PCB 153 concentration ranging from 2 to 9%, depending on the species of marine biota and location; a similar trend was also observed in PCB 118.

**Fig. 3:**
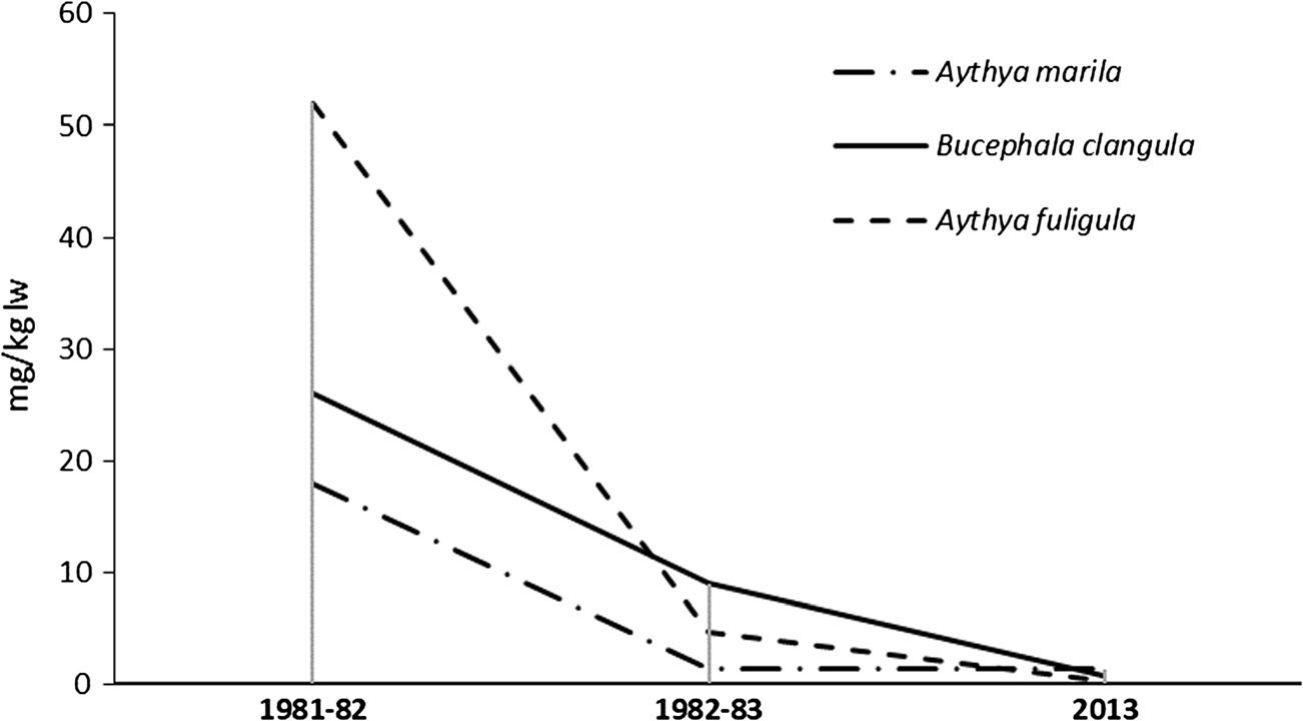
Temporal differences in PCB residues

Clear trends can be observed in the concentrations of contaminants tested in the present study in ducks wintering in the southern Baltic Sea (Fig. 3). The current PCB levels in the fat tissue of tufted duck, greater scaup, and common goldeneye were compared with those given in data from 1981 to 1983 (Falandysz and Szefer 1988). The greatest differences were found for the tufted duck, with mean Σi-PCB concentrations in the fat tissue varying from 52 ± 19 mg/kg lw in birds caught in winter 1981–1982, 4.6 mg/kg lw a year later, and 191.80 ng/g lw (0.192 mg/kg) in 2013. The average reduction rate in Σi-PCB concentration in the fat tissue of tufted ducks wintering in the Baltic Sea southern coast was estimated at 3.3% per year. For the common goldeneye, Σi-PCB concentration fell from 26 mg/kg lw in 1981–1982 to 9 mg/kg lw in 1982–1983, and then to 0.646 mg/kg lw in 2013; in this case, the mean decrease was 3.1% per year; in the greater scaup, the decrease was 2.9% per year. When assessing temporal changes in PCB concentration in duck tissues, it is necessary to also consider the underestimation of PCB concentration in these studies, as indicated by SRM recovery (Table 3), and the differences in analytical selectivity between the 1980s and the present. Hence, it is impossible to rule out that the observed difference in PCB concentration may actually be smaller than observed.

## Conclusions

Reference data indicates that a systematic decrease in PCB concentration in the Baltic Sea region can still be observed in both bottom sediments and marine animal tissues. Our findings show that animals related to marine habitats also demonstrate decreasing tendencies in PCB residues through trophic chains, although the observed differences in PCB concentrations could be influenced by differences in analytical selectivity and the fact that the obtained results may be underestimated. Although insufficient data exists regarding PCB residues in the tissues of ducks wintering in the Baltic Sea coasts to unequivocally interpret our results, the collected body of evidence nevertheless affords an insight into the level of PCB pollution in the Baltic Sea. Despite the fact that the noted PCB concentrations in duck tissues were sometimes lower than reported by other authors and for other bird species and water areas, our analysis of PCB residues clearly indicates that the common pochard and tufted duck are not suitable for human consumption, due to the high Σi-PCB concentrations present. In turn, the regular consumption of muscle and liver of game birds does not appear to cause unacceptable dl-PCB intake above the TWI value.
